# Emotional intelligence in patients with psoriasis and atopic dermatitis: Impaired integration of emotions and decision-making

**DOI:** 10.1192/j.eurpsy.2021.268

**Published:** 2021-08-13

**Authors:** O. Belugina

**Affiliations:** Psychiatry And Medical Psychology, Belarusian State Medical University, Minsk, Belarus

**Keywords:** Emotional intelligence, psoriasis, atopic dermatitis, social relations

## Abstract

**Introduction:**

Emotional intelligence (EI) is a fundamental requirement to maintaining social activity. Patients with psoriasis and atopic dermatitis have difficulties in emotional awareness.

**Objectives:**

The objective of this study is to assess EI in patients with atopic dermatitis and psoriasis.

**Methods:**

Patients with psoriasis n=67, atopic dermatitis n=59 and control group n=65 were included in cross-sectional study. EI and its main components (experiential: perceiving emotions and using emotions to facilitate thought; strategic: understanding emotions and managing emotions to promote personal growth and social relations) were assessed using The Mayer–Salovey–Caruso Emotional Intelligence Test 2.0. Statistical analyses were performed using One-Way ANOVA and One-Way ANOVA (Kruskal-Wallis test). The level of statistical significance was set at p<0.05.Data are presented as the Me (±SD).

**Results:**

Our results show that there is statistically significant lower “strategic” component of EI for psoriasis Me=0.367 (±0.0455) and atopic dermatitis Me=0.369 (±0.0353) than for the control group Me= 0.381(±0.0361), (χ2 =7.15; p= 0.028). “Managing emotions to promote personal growth and social relations” is presented with statistically significant lower for psoriasis Me= 0,293 (±0.0374) and atopic dermatitis Me= 0.301 (±0.0351) than for the control group Me= 0.312 (±0.0272), (F=0.05; p=0.007). There is no statistically significant difference between other components of EI and the EI scores in three groups.

**Conclusions:**

Patients with psoriasis and atopic dermatitis have emotional difficulties when it comes to making effective decisions.

**Disclosure:**

No significant relationships.

